# Antipsychotic prescribing patterns in children and adolescents attending Australian general practice in 2011 and 2017

**DOI:** 10.1002/jcv2.12208

**Published:** 2023-11-10

**Authors:** Julie Klau, David Gonzalez‐Chica, Melissa Raven, Jon Jureidini

**Affiliations:** ^1^ Robinson Research Institute Critical and Ethical Mental Health Research Group University of Adelaide Adelaide South Australia Australia; ^2^ Discipline of General Practice University of Adelaide Adelaide South Australia Australia

**Keywords:** adolescents, antipsychotic prescribing, children, off‐label, polypharmacy

## Abstract

**Background:**

Antipsychotics are increasingly prescribed to children and adolescents worldwide, but little is known about reasons for prescribing. We aimed to examine patterns of paediatric antipsychotic prescribing in Australian primary care services in 2011 and 2017, including diagnoses, sociodemographic characteristics, off‐label prescribing, and psychotropic co‐prescribing.

**Methods:**

Retrospective analysis of electronic health records (EHRs) using a large Australian general practice database (MedicineInsight). Diagnoses of mental disorders were extracted from EHRs and associated with antipsychotic prescriptions within the same calendar year for three age‐groups: 0–9, 10–14, and 15–18‐year‐olds.

**Results:**

In 2017, children/adolescents with mental health diagnoses were more likely to be prescribed antipsychotics (2.9% of 27,412 patients) than in 2011 (2.0% of 8418 patients; absolute difference +0.9, 95% CI + 0.5, +1.4). The likelihood was greater for patients with bipolar disorders (21.6% vs. 41.5%), eating disorders (1.1% vs. 7.2%), and autism without behavioural problems (3.7% vs. 6.1%). Depression/anxiety (adjusted 26.8% of patients 2011; 30.8% 2017) was the most common diagnosis associated with antipsychotics in both years. Most antipsychotics were prescribed off‐label (69.8% 2011; 79.7% 2017; absolute difference +9.8, 95% CI + 1.54, +18.4). Off‐label prescribing increased most among those aged 15–18‐years, females, and patients living in outer regional/remote/very remote communities and the most disadvantaged areas. The three most frequently prescribed antipsychotics in both years were risperidone, quetiapine, and olanzapine. Psychotropic co‐prescribing among patients receiving antipsychotic prescriptions was approximately 69% in both years.

**Conclusions:**

Prescribing antipsychotics for mental health diagnoses to children/adolescents attending Australian general practices was more frequent in 2017 than 2011, and most commonly associated with depression/anxiety diagnoses. In both years, most prescribing was off‐label. The majority of patients were co‐prescribed other classes of psychotropics along with antipsychotics.

## Graphical abstract

Children and adolescents were more likely to be prescribed antipsychotics for mental health conditions in 2017 than 2011, despite guidelines recommending non‐drug treatments wherever possible. Off‐label prescribing was common, reaching 80% in 2017. Antipsychotics were mostly associated with diagnoses of anxiety and depression. In 2017 74% of patients were prescribed another psychotropic medication along with an antipsychotic.

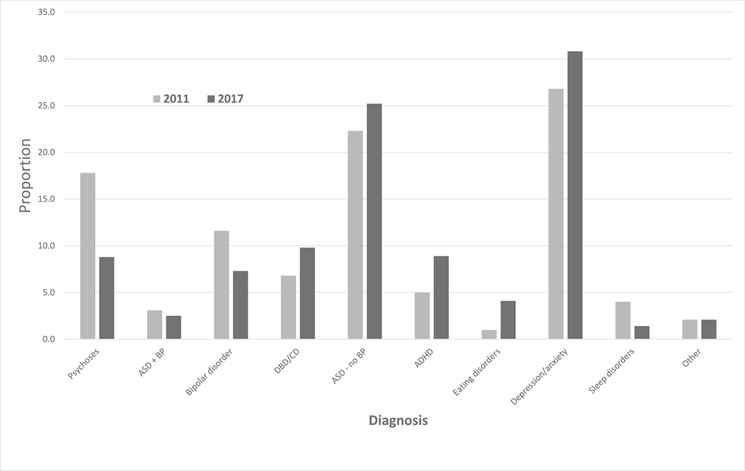

**Key points**

- Previous research shows high rates of off‐label prescribing of antipsychotics to children/adolescents
- Most prescribing by Australian GPs appears to be off‐label, and increasing—(70% 2011—80% 2017)
- The main diagnosis among children/adolescents prescribed antipsychotics was depression/anxiety
- Co‐prescribing of other psychotropics was frequent (70% of patients)
- More support is needed for Australian GPs to improve antipsychotic prescribing practices to children/adolescents, and make alternative treatments more accessible.

## INTRODUCTION

Antipsychotics are increasingly being prescribed to children and adolescents worldwide (Bachmann et al., 2014; Hálfdánarson et al., 2017; Klau et al., 2022) despite ongoing controversy over their use in paediatric patients (De Hert et al., 2011). Off‐label prescribing is also common, as evidenced in a systematic review (Carton et al., 2015), in which 36%–93% of paediatric antipsychotic prescribing was off‐label. Off‐label prescribing is the prescribing of drugs for medical conditions for which they have not been approved by regulatory authorities (off‐label by indication), as well as prescribing drugs for age‐groups for which they have not been approved (off‐label by age), which is particularly common in children/adolescents (Sharma et al., 2016). In the United States, in a study of office‐based visits to psychiatrists and non‐psychiatrist physicians from 2005 to 2009, 94.0% and 87.3% of children and adolescents, respectively, had no Food and Drug Administration (FDA) approved indication recorded for antipsychotic prescriptions (Olfson et al., 2012).

Studies in the United States (Edelsohn et al., 2017; Penfold et al., 2013) have found antipsychotics were predominantly prescribed to children/adolescents for attention deficit hyperactivity disorder (ADHD) and disruptive or aggressive behaviours. As age increased, antipsychotics were increasingly prescribed for depression in several U.S. studies (Edelsohn et al., 2017; Olfson et al., 2015) and a Finnish study (Varimo et al., 2020).

However, little is currently known about antipsychotic prescribing patterns in children/adolescents in Australia, nor the extent to which prescribing conforms with approvals by the Therapeutic Goods Administration (TGA), a key regulator in the Australian healthcare system (see Appendix 1). In Australia, some studies (e.g., Karanges et al., 2014) have examined psychotropic medication data from the Pharmaceutical Benefits Scheme (PBS), which provides information on prescriptions of subsidised medications, but no information on clinical indications.

Our literature search retrieved only four Australian studies that provided information about diagnoses. Firstly, an audit of case‐notes at the Women's and Children's Hospital in South Australia in 2012 found most antipsychotic prescriptions were for children with intellectual disabilities or autism with behavioural problems (Ellis & Angley, 2013). The second study examined reasons for antipsychotic prescribing to 12–17‐year‐old attendees at a public mental health clinic in New South Wales (Dharni & Coates, 2018). Twenty‐four precent of patients were prescribed antipsychotics, mostly with antidepressants, and primarily for anxiety disorders or sleep issues. However, in that study, patients with psychoses were excluded, and for missing diagnoses, reason for presentation was used as a proxy, casting doubt on the accuracy of diagnoses. The third and fourth studies, both retrospective file reviews, examined prescribing of all psychotropics in a Western Australian eating disorders inpatient unit (Moore et al., 2013), and a Brisbane child and youth mental health service (Dean et al., 2006). The main reasons for prescribing antipsychotics were agitation and/or anxiety (Moore et al., 2013) and behavioural disturbances (Dean et al., 2006). In all studies, the sample size was small and unrepresentative of prescribing in the wider community.

The aims of this study are to examine (1) proportions of children/adolescents with mental health diagnoses who received antipsychotic prescriptions, across time and in different socioeconomic levels, age‐groups and geographical locations, (2) most frequent diagnoses associated with antipsychotic prescriptions, (3) off‐label prescribing frequencies, (4) most frequently prescribed antipsychotics, and (5) frequencies of co‐prescription with other classes of psychotropic drugs.

## METHODS

### Study population and data source

This study analysed electronic health records (EHRs) of children/adolescents under 19 years attending Australian general practices in 2011 and 2017, using MedicineInsight, a large primary care database established in 2011. Australia has a universal healthcare system, with a mix of public and private healthcare services (Appendix 1). Patients covered by Medicare are entitled to subsidised medical services from the Medicare Benefits Scheme (MBS), and a wide range of medicines approved on the Pharmaceutical Benefits Scheme (PBS). General practitioners (GPs; family physicians) provide more MBS‐subsidised mental health care than any other providers, with 38% of consultations in a typical week addressing mental health concerns (Thornley & Harris, 2021). Psychotropic drugs, with the exception of stimulants, are mostly prescribed by GPs (www.aihw.gov.au/mental‐health/topic‐areas/mental‐health‐prescriptions).

In the 2018/2019 financial year, a total of 569 general practices and 3255 general practitioners (GPs; family physicians) were registered with MedicineInsight, representing 7% of all practices and 9% of all GPs (NPS MedicineWise, 2020a). On joining MedicineInsight, practices have whole‐of‐practice de‐identified clinical records, including patient sociodemographics, uploaded to a data warehouse using a secure third‐party extraction tool (Havard et al., 2021). Information on all clinical encounters, including medications prescribed, diagnoses, medical tests and results, and reasons for encounters, are uploaded monthly, generating an ongoing record of patient encounters (Havard et al., 2021).

To improve data quality, only practices with at least 2 years of data and no interruptions of more than 6 weeks are provided to researchers by MedicineInsight. Additionally, we excluded practices with substantial variations in patient numbers over time (i.e., over five times higher in 2018 than 2011). 402 general practices met the quality standards for inclusion, and had antipsychotic prescriptions recorded for children or adolescents within the study period.

As a further quality measure, only patients classified as ‘regular patients’, at any time during the study period, were included. Regular patients were defined as those who attended a specific general practice at least three times within 2 years, with at least one visit each year. Patients may attend other practices outside and within MedicineInsight, resulting in some incomplete treatment records and some duplication of patients, the latter estimated as less than 4% in the database (NPS MedicineWise, 2020b). Including only regular patients maximises the likelihood that patient records are a more complete record of their attendance. As a sensitivity analysis, we also performed an analysis without restricting the sample to regular patients.

The final sample included 168,009 children/adolescents (regular patients) in 2011 and 301,643 in 2017.

### Study variables

#### Antipsychotic prescriptions

We included recorded prescriptions of all antipsychotics listed in the Anatomical Therapeutic Chemical (ATC) index (World Health Organization [WHO], 2023), and available in Australia. A list of antipsychotics and indications approved by the TGA for use in children/adolescents in Australia is provided in Supporting Information Table S1.

#### Mental health diagnoses

GPs record health conditions using a drop‐down list in EHR practice software and/or free‐text fields in three variable fields within MedicineInsight: diagnosis, reason for visit, and reason for prescription. We used coding algorithms to extract diagnostic information based on keywords relating to mental health diagnoses (Supporting Information Table S2). In some cases, terms used in the algorithms represent symptoms; but, for mental health diagnoses, they are often also used to represent disorders (e.g., anxiety) (Havard et al., 2021). A mental health diagnosis was recorded for the patient if the algorithm identified the corresponding term/diagnosis in at least one of the three fields. This procedure for extracting diagnoses was validated by Havard et al. A recorded diagnosis was associated with an antipsychotic prescription if the diagnosis was recorded from 180 days before through to 90 days after the prescription, or both occurred within the same calendar year.

We used a hierarchical approach, adapted from Zito et al. (2013), to determine the most likely diagnoses for patients prescribed antipsychotics. Higher‐level categories represent conditions with stronger clinical rationales for use of antipsychotics, based on existing clinical guidelines and Australian regulatory approvals. Ten mutually‐exclusive levels were identified: psychoses, including schizophrenia; autism with behavioural problems; bipolar disorder; disruptive behaviour/conduct disorder (DBD/CD); autism without behavioural problems; ADHD; eating disorders; depression/anxiety; sleep disorders; and ‘other’ (post‐traumatic stress disorder [PTSD], obsessive‐compulsive disorder, personality disorders, communication disorders [language, learning], substance use disorders, enuresis/incontinence disorders).

Our preliminary analyses found 154 patients prescribed antipsychotics without associated mental health diagnoses in 2011, and 392 in 2017 (Supporting Information Table S3). A small percentage of these patients received prescriptions for approved neurological conditions such as Tourette's and epilepsy (7.1%, 2011; 3.6% 2017). A larger minority of patients had mental health plans or suicidal behaviours recorded, without specific mental health diagnoses (13.6%, 2011; 28.6%, 2017). However, the majority had no associated psychiatric or neurological reason for prescribing (79.2%, 2011; 68.4%, 2017). Some may have been diagnosed by paediatricians or child/adolescent psychiatrists, and the diagnostic records not captured in the dataset, or the diagnoses may have been recorded in doctors' progress notes, which, for confidentiality reasons, are not included in the database. In these circumstances, our diagnosis algorithm would associate non‐psychiatric reasons with antipsychotic prescriptions. We cannot be confident that these reasons would provide a good representation of the primary reasons for prescribing. Therefore, we restricted our analyses to the subsample with mental health diagnoses.

#### Off‐label prescribing

Prescription of antipsychotics was considered off‐label if the drug was not approved by the TGA for the condition identified in the hierarchy of diagnoses and the age of the child. The algorithm for identifying off‐label prescribing is provided in Supporting Information Table S4. A combined off‐label variable for all prescriptions across all diagnoses was created. If more than one antipsychotic was prescribed in the calendar year, the diagnosis was considered to have an on‐label antipsychotic if any of the prescriptions was on‐label for the diagnosis.

#### Sociodemographic factors

We analysed gender, age, rurality and socioeconomic status. Gender was divided into male and female, and age was categorised into three groups: 0–9, 10–14, and 15‐18‐years. Rurality of patient residence was categorised as major cities, inner regional, or outer regional/remote/very remote, using the Australian Statistical Geography Standard (ASGS) (Australian Bureau of Statistics [ABS], 2016b). Socioeconomic status was analysed using the Index of Relative Socio‐economic Advantage and Disadvantage (IRSAD), divided into quintiles. IRSAD is an indicator based on residential postcodes, developed by the Australian Bureau of Statistics using census data on income, education, employment status, and housing (Australian Bureau of Statistics [ABS], 2016a). If patient IRSAD was missing, practice IRSAD was used as a proxy.

#### Psychotropic co‐prescribing

Psychotropic co‐prescribing was defined as writing a prescription for at least one other class of psychotropic on the same day an antipsychotic was prescribed. The other medication classes, detailed in an earlier study by our team (Klau et al., 2022), were antidepressants, ADHD medications (stimulants, atomoxetine and alpha‐2 agonists, which are approved in Australia for ADHD), anxiolytics and hypnotics/sedatives, with melatonin examined separately.

### Statistical analysis

Proportions of patients with mental health diagnoses with associated antipsychotic prescriptions, and proportions receiving off‐label prescriptions, were calculated for 2011 and 2017. Frequencies of associated diagnoses were also calculated. Results were adjusted for patient age, gender, and IRSAD, and practice characteristics (rurality, state, IRSAD). Differences in proportions from 2011 to 2017 were analysed using logistic regression, reporting marginal predicted percentages derived from odds ratios, with 95% confidence intervals, using practice as a cluster and robust standard errors. Differences according to sociodemographic characteristics were also analysed using a similar approach. Unadjusted percentages were calculated for the most frequently prescribed antipsychotics for each diagnostic group and for psychotropic co‐prescribing.

Stata version 16 (StataCorp) was used for all analyses.

## RESULTS

### Sample characteristics

In 2011, 8418 of 168,009 (4.2%) children/adolescents had mental health diagnoses recorded (Table 1). In 2017 there were 27,412 of 301,643 (6.7%) (adjusted difference +2.5%, 95% CI [2.2, 2.8], *p* < 0.001; Supporting Information Table S5).

**TABLE 1:** Sample characteristics.

	Total sample 0‐18 year‐olds		Sample with mental health diagnosis		Sample with mental health diagnosis prescribed antipsychotic	
	2011	2017	2011	2017	2011	2017
N	168,009	301,643	8418	27,412	191	893
Age group						
<10 years	57.1%	58.4%	44.2%	41.5%	15.2%	12.0%
10–14 years	23.9%	21.9%	23.4%	25.3%	27.2%	27.3%
15–18 years	19.0%	19.7%	32.4%	33.3%	57.6%	60.7%
Gender						
Male	48.0%	50.0%	53.3%	53.3%	57.6%	53.4%
Female	52.0%	50.0%	46.7%	46.7%	42.4%	46.6%
IRSAD						
1. Most advantaged	28.1%	28.0%	24.7%	25.3%	13.6%	16.4%
2	18.3%	18.5%	18.0%	18.1%	16.2%	13.8%
3	21.9%	21.9%	22.6%	23.2%	22.5%	26.0%
4	14.2%	14.9%	16.5%	16.8%	20.9%	23.0%
5. Least advantaged	17.6%	16.7%	18.1%	16.6%	26.7%	20.9%
Remoteness						
Major cities	61.4%	62.8%	60.0%	60.9%	46.1%	48.3%
Inner regional	25.2%	24.4%	29.5%	28.8%	40.3%	37.2%
Outer regional/remote/very remote	13.4%	12.8%	10.6%	10.3%	13.6%	14.5%

Abbreviation: IRSAD, Index of Relative Socio‐economic Advantage and Disadvantage.

191 children/adolescents with mental health diagnoses were prescribed antipsychotics in 2011, and 893 in 2017.

In both years, children 0‐9‐years (approximately 58% of the total sample) were the most likely to receive any mental health diagnosis, but the least likely to receive antipsychotic prescriptions for a diagnosis (Table 1). Of the patients with mental health diagnoses receiving antipsychotic prescriptions, most resided in major cities (46.1%, 2011; 48.3%, 2017) or inner regional areas (40.3%, 2011; 37.2%, 2017). In both years, most patients who received antipsychotic prescriptions for their diagnoses, were male (57.6%, 2011; 53.4%, 2017). The distribution by IRSAD was similar both years, with most patients receiving prescriptions (70%) living in the lower three IRSAD quintiles (most disadvantaged).

### Diagnoses

A higher proportion of patients with mental health diagnoses were given antipsychotic prescriptions in 2017 (2.0% in 2011; 2.9% in 2017, *p* < 0.001; Table 2).

**TABLE 2:** Proportions of children and adolescents with mental health diagnoses prescribed antipsychotics, 2011 and 2017.

	2011		2017		Absolute difference 2017–2011	
	*N* with diagnosis	Adjusted^b^ % of diagnostic group receiving antipsychotic prescriptions	*N* with diagnosis	Adjusted^b^ % of diagnostic group receiving antipsychotic prescriptions	% (95% CI)	*p* value
All diagnoses^a^	8418	2.0	27,412	2.9	0.9 (0.5, 1.4)^b^	<0.001
Psychoses, including schizophrenia	67	45.7	209	34.0	−11.7 (−25.8, 2.5)	0.105
Autism with behavioural problems	63	10.8	244	8.9	−1.9 (−9.5, 5.7)	0.616
Bipolar disorder	82	21.6	156	41.5	19.9 (8.7, 31.0)	<0.001
Disruptive behaviour/conduct disorder	852	1.3	2798	2.9	1.6 (0.4, 2.9)	0.011
Autism without behavioural problems	1100	3.7	3086	6.1	2.4 (0.2, 4.5)	0.030
ADHD	893	0.9	3517	1.8	0.9 (0.1, 1.7)	0.022
Eating disorders	182	1.1	523	7.2	6.1 (3.1, 9.1)	<0.001
Depression or anxiety	2872	1.5	10,183	2.5	1.0 (0.4, 1.5)	0.001
Depression	1623	1.9	4710	3.9	2.0 (1.2, 2.9)	<0.001
Anxiety	1748	1.4	7776	2.1	0.8 (0.1, 1.4)	0.030
Depression and anxiety	499	2.1	2303	4.5	2.4 (1.0, 3.8)	0.001
Sleep disorders	729	0.9	2630	0.4	−0.4 (−1.1, 0.3)	0.210
Other mental health^c^	1578	0.2	4066	0.4	0.3 (−0.1, 0.5)	0.099

Abbreviation: ADHD, attention deficit hyperactivity disorder.

^a^ Only one diagnosis is recorded per patient according to hierarchy. Highest level (1: psychosis) through to lowest level (10: other). Diagnoses higher in hierarchy have stronger clinical rationale for use of antipsychotics. Numbers adjusted for age, gender, patient IRSAD, and practice characteristics (state, rurality, practice IRSAD).

^b^ Analysis using logistic regression adjusted for age, gender, patient IRSAD, and practice characteristics (state, rurality, practice IRSAD). IRSAD, Index of Relative Socio‐economic Advantage and Disadvantage.

^c^ ‘Other’ includes obsessive‐compulsive disorder, post‐traumatic stress disorder, personality disorder, substance use disorder, incontinence disorder, including bedwetting, learning and language disorders.

Higher prescribing proportions were observed in 2017 for patients diagnosed with bipolar disorder, autism without behavioural problems, depression/anxiety or behavioural disorders (ADHD, DBD/CD). However, eating disorders had the largest difference of all diagnoses (6.5 times more likely to be prescribed antipsychotics in 2017 than in 2011). For some analyses, however, numbers were low, limiting the reliability of the findings.

**FIGURE 1:**
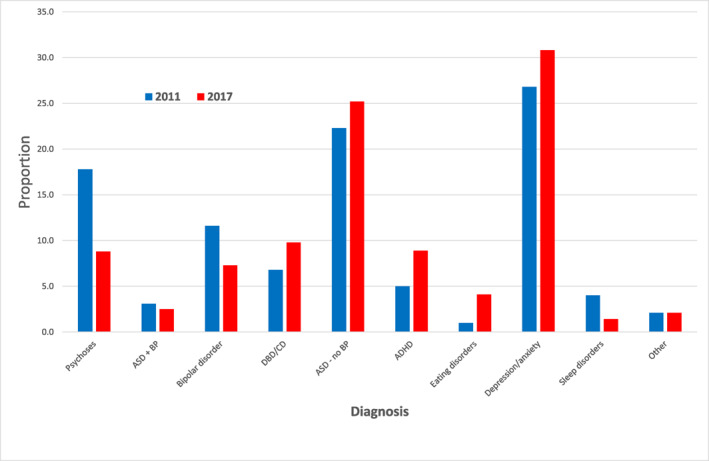
Main diagnosis among children and adolescents receiving antipsychotic prescriptions, 2011 and 2017. Only one diagnosis is recorded per patient according to hierarchy. Highest level (1: psychosis) through to lowest level (10: other). Diagnoses higher in hierarchy have stronger clinical rationale for use of antipsychotics. Numbers adjusted for age, gender, patient IRSAD, and practice characteristics (state, rurality, practice IRSAD). ‘Other’ includes obsessive‐compulsive disorder, post‐traumatic stress disorder, personality disorder, substance use disorder, incontinence disorders (including enuresis/bed wetting), learning disorder, language disorder. ADHD, attention deficit hyperactivity disorder; ASD, autism spectrum disorder; BP, behavioural problems; CD, conduct disorder; DBD, disruptive behaviour disorder; IRSAD, Index of Relative Socio‐economic Advantage and Disadvantage.

Analysing diagnoses associated with antipsychotic prescriptions (Figure 1), showed most patients in 2011 had diagnoses of depression/anxiety (26.8%), followed by diagnoses of autism without behavioural problems (22.3%) and psychoses (17.8%). A similar pattern was found in 2017, with percentages of 30.8% (depression/anxiety) and 25.2% (autism without behavioural problems), but DBD/CD replaced psychoses in third place (9.8%).

### Off‐label prescribing

Table 3 presents the results for off‐label prescribing among patients with mental health diagnoses who received antipsychotic prescriptions. The proportion of off‐label prescribing was higher in 2017 (79.7%) than 2011 (69.8%), with the most marked increases occurring in 15‐18‐year‐olds, females, those living in outer regional/remote/very remote areas or the most disadvantaged areas (lowest IRSAD quintile). Analysing by diagnosis, psychotic disorders had a higher frequency of off‐label prescribing in 2017 (46.7% 2017 vs. 16.3% 2011; +30.4, 95% CI [15.4, 45.5], *p* < 0.001). Numbers were too low to reliably examine changes in other disorders for which antipsychotic approvals existed (bipolar disorder, behavioural problems in autism or DBD/CD). The higher level of off‐label prescribing in 2017 was mostly attributable to diagnoses of autism without behavioural problems and diagnoses of depression/anxiety.

**TABLE 3:** Proportions of children and adolescents receiving off‐label antipsychotic prescriptions by sociodemographic characteristics, 2011 and 2017.

	Adjusted prevalence^a^ 2011 (%)	Adjusted prevalence^a^ 2017 (%)	Overall absolute difference 2017–2011 (95% CI)^a^	*p* value
Total	69.8	79.7	10.0 (1.5, 18.4)	0.020
Age groups				
0–9 years	84.4	82.6	−1.8 (−17.7, 14.1)	0.827
10–14 years	**86.4**	81.1	−5.3 (−16.5, 5.9)	0.350
15–18 years	56.6	78.2	21.6 (10.4, 32.8)	<0.001
Gender				
Male	71.4	75.5	4.1 (−6.6, 14.8)	0.449
Female	66.9	**84.8**	17.8 (7.0, 28.6)	0.001
Rurality				
Major cities	76.7	78.4	1.6 (−9.2, 12.5)	0.767
Inner regional	69.6	80.0	10.4 (−3.6, 24.4)	0.145
Outer regional/remote/very remote	48.4	82.7	34.3 (15.1, 53.4)	<0.001
IRSAD quintile^b^				
1. Least disadvantaged	78.7	87.2	8.4 (−8.6, 25.4)	0.333
2	75.9	80.8	4.9 (−10.7, 20.5)	0.537
3	73.7	77.0	3.4 (−14.1, 20.8)	0.705
4	70.4	80.2	9.8 (−9.4, 28.9)	0.318
5. Most disadvantaged	57.7	77.4	19.7 (1.9, 37.6)	0.030

*Note*: Numbers marked in bold type indicate *p* < .05 (Bonferroni adjusted) for the difference between each category and the reference category for the given year. Reference categories were: 15–18 years, male, major cities and IRSAD quintile1 (Least disadvantaged).

^a^ Analyses using logistic regression adjusted for age, gender, patient IRSAD, and practice characteristics (state, rurality, practice IRSAD), and reporting predicted probabilities.

^b^ IRSAD, Index of Relative Socio‐economic Advantage and Disadvantage.

### Sociodemographic characteristics of children/adolescents prescribed antipsychotics

Adjusted analyses comparing frequencies of antipsychotic prescribing to children/adolescents with mental health diagnoses for 2011 and 2017, by sociodemographic characteristics, are presented in Table 4. In most sociodemographic groups, the proportion of patients with mental disorders prescribed antipsychotics was higher in 2017. The greatest differences were for 15–18‐year‐olds (3.6%, 2011 vs. 5.5%, 2017), those living in outer regional/remote/very remote areas (2.1% vs. 3.6%) and in the middle IRSAD quintile (2.3% vs. 3.7%). The male/female ratio declined from 1.8 in 2011 to 1.5 in 2017.

**TABLE 4:** Proportions of children and adolescents with mental health diagnoses prescribed antipsychotics, by sociodemographics, 2011 and 2017.

	2011		2017		Absolute difference 2017–2011	
	Number with mental health diagnosis	Diagnoses prescribed AP (%)^a^	Number with mental health diagnosis	Diagnoses prescribed AP (%)^a^	% (95% CI)^a^	*p* value
Total	8418	2.0	27,412	2.9	0.9 (0.5, 1.4)	<0.001
Age group						
0–9 years	3722	**0.6**	11,366	**0.9**	0.3 (−0.2, 0.8)	0.270
10–14 years	1971	**2.2**	6926	**3.1**	0.9 (0.03, 1.7)	0.043
15–18 years	2725	3.6	9120	5.5	1.8 (1.0, 2.7)	<0.001
Gender						
Male	4486	2.6	14,603	3.5	0.9 (0.2, 1.6)	0.016
Female	3928	**1.4**	12,791	**2.4**	0.9 (0.5, 1.4)	<0.001
Rurality						
Major cities	5049	1.7	16,695	2.5	0.8 (0.2, 1.4)	0.012
Inner regional	2481	2.4	7881	3.3	0.9 (0.1, 1.7)	0.024
Outer regional/remote/very remote	888	2.1	2836	3.6	1.5 (0.4, 2.6)	0.006
IRSAD quintile						
1. Least disadvantaged	2076	1.1	6929	1.9	0.8 (0.2, 1.3)	0.006
2	1516	2.1	4967	2.6	0.4 (−0.5, 1.4)	0.341
3	1904	2.3	6349	**3.7**	1.5 (0.4, 2.5)	0.005
4	1392	2.2	4614	3.4	1.2 (0.2, 2.2)	0.019
5. Most disadvantaged	1526	2.3	4543	3.0	0.7 (−0.1, 1.5)	0.097

*Note*: Numbers marked in bold type indicate *p* < .05 (Bonferroni adjusted) for the difference between each category and the reference category for the given year. Reference categories were: 15–18 years, male, major cities and IRSAD quintile1 (Least disadvantaged).

Abbreviations: AP, antipsychotic; IRSAD, Index of Relative Socio‐economic Advantage and Disadvantage.

^a^ Analyses using logistic regression adjusted for age, gender, patient IRSAD, and practice characteristics (state, rurality, practice IRSAD), and reporting predicted probabilities.

### Specific antipsychotics

For both years, the three most frequently prescribed antipsychotics were risperidone, quetiapine and olanzapine (Table 5). Risperidone was the most frequent in 2011 (48.8% of all prescriptions), and quetiapine (41.4%) in 2017. In both years, quetiapine was the most commonly prescribed antipsychotic for psychoses (37.2%, 2011; 33.0%, 2017), bipolar disorder (50.0%; 58.2%) and depression/anxiety (72.0%; 76.2%), but risperidone was the most commonly prescribed antipsychotic for autism, ADHD and disruptive behaviours. Olanzapine constituted 41.7% of antipsychotic prescriptions for eating disorders in 2017.

**TABLE 5:** Most prescribed antipsychotics associated with each diagnostic group, with clinical hierarchy^a^, 2011 and 2017.

	2011					2017				
	AP total	*N* patients	AP type	*n*	%^b^	AP total	*N* patients	AP type	*n*	%^b^
All	209	191	RIS	102	48.8	963	893	QUE	399	41.4
			QUE	72	34.4			RIS	368	38.2
			OLAN	21	10.0			OLAN	103	10.7
Psychoses, including schizophrenia	43	33	QUE	16	37.2	103	81	QUE	34	33.0
			RIS	14	32.6			OLAN	24	23.3
			OLAN	10	23.3			ARIP	15	14.6
Autism with behavioural problems	7	7	RIS	7	100.0	26	23	RIS	19	73.1
								OLAN	<5	‐
								QUE	<5	‐
Bipolar disorder	24	21	QUE	12	50.0	79	66	QUE	46	58.2
			RIS	<5	‐			OLAN	13	16.5
			OLAN	<5	‐			RIS	8	10.1
Disruptive behaviour/conduct disorders	13	13	RIS	11	84.6	80	76	RIS	60	75.0
			QUE	<5	‐			QUE	9	11.3
								OLAN	5	6.3
Autism spectrum disorders	47	44	RIS	42	89.4	217	206	RIS	177	81.6
			PAL	<5	‐			QUE	18	8.3
			QUE/PER	<5	‐			ARIP	10	4.6
Attention deficit hyperactivity disorders	10	10	RIS	8	80.0	79	78	RIS	57	72.2
			QUE/PER	<5	20.0			QUE	14	17.7
								ARIP	<5	‐
Eating disorders	<5	<5	RIS	<5	‐	36	34	QUE	20	55.6
			OLAN	<5	50.0			OLAN	15	41.7
								RIS	<5	‐
Depression/anxiety	50	50	QUE	36	72.0	311	300	QUE	237	76.2
			RIS	14	28.0			OLAN	31	10.0
			OLAN	10	20.0			RIS	26	8.4
Sleep disorders	8	7	RIS	<5	‐	14	14	QUE	10	71.4
			QUE	<5	‐			RIS	<5	‐
			OLAN/HAL	<5	‐			OLAN/HAL	<5	‐
Other^c^	5	<5	RIS	<5	‐	18	15	QUE	9	50.0
			QUE	<5	‐			RIS	<5	‐
								OLAN	<5	‐

*Note*: Numbers and percentages omitted where cell size <5 patients.

Abbreviations: AP, antipsychotic; ARIP, aripiprazole; HAL, haloperidol; MEL, melatonin; OLAN, olanzapine; PER, periciazine; QUE, quetiapine; RIS, risperidone.

^a^ Only one diagnosis is recorded per patient according to hierarchy. Highest level (1: psychosis) through to lowest level (10: other). Diagnoses higher in hierarchy have stronger clinical rationale for use of antipsychotics.

^b^ Percentages reported as unadjusted proportion of all patients on antipsychotics for each diagnostic group. Patients may have had more than one co‐prescribed medication class.

^c^ ‘Other’ includes obsessive‐compulsive disorder, post‐traumatic stress disorder, personality disorder, substance use disorder, incontinence disorder, including bed‐wetting, learning disorder, and language disorders.

### Psychotropic co‐prescribing

Co‐prescribing of other classes of psychotropic drugs was common (Supporting Information Table S6). In adjusted analyses, 69.7% of patients in 2011 and 69.4% in 2017 were prescribed different classes of psychotropics the same day as antipsychotics. In both years, antidepressants were the most co‐prescribed psychotropic medications for most disorders, followed by ADHD medications. The third most frequently prescribed concurrent medication changed from anxiolytics in 2011 to melatonin in 2017.

### Sensitivity analyses

Table 1 was replicated without restricting the sample to regular patients. The results are presented in Supporting Information, Table S7. Results were near identical, with very minor changes in predicted proportions, confidence intervals and *p*‐values.

## DISCUSSION

This study analysed patterns of antipsychotic prescribing to children/adolescents in Australian primary care in 2011 and 2017. There are three notable findings. Firstly, compared with 2011, patients with any mental health diagnosis in 2017 were more likely to be prescribed antipsychotics. Although frequencies of antipsychotic prescribing remained relatively stable for psychoses, autism with behavioural problems, and sleep disorders, patients with other diagnoses were more likely to be prescribed antipsychotics in 2017. Secondly, off‐label prescribing was higher in 2017 (79.7%) than 2011 (69.8%), especially for females, 15‐18‐year‐olds, and patients living in disadvantaged areas and remote geographical regions. Thirdly, in both years, almost 70% of patients prescribed antipsychotics were co‐prescribed other classes of psychotropics.

### Diagnostic patterns associated with antipsychotic prescriptions

Consistent with international (Carton et al., 2015) and Australian (Dharni & Coates, 2018; Ellis & Angley, 2013) findings, we observed high levels of off‐label prescribing, increasing from 69.8% in 2011 to 79.7% in 2017.

Most patients who were prescribed antipsychotics had off‐label diagnoses of depression/anxiety, with comparatively low frequencies of prescribing associated with ADHD. This contrasts with international findings. Several studies examining antipsychotic prescription rates across all prescribers, and reporting diagnoses with hierarchy (Crystal et al., 2009), and without hierarchy in the United States (Edelsohn et al., 2017; Olfson et al., 2015), and Germany (Bachmann et al., 2014), have found ADHD was one of the two most frequent diagnoses. The difference likely reflects differing regulatory landscapes. ADHD patients in Australia are generally prescribed stimulants by specialists (Australian ADHD Professionals Association, 2023). Although GPs can co‐manage treatment of children with ADHD, complex cases likely to be considered for non‐routine medications such as antipsychotics may be solely managed by paediatricians or child psychiatrists. Even so, despite being low in number, the proportion of patients with ADHD prescribed antipsychotics in primary care doubled from 2011 to 2017.

Several studies have reported antipsychotic prescription frequencies associated with depression/anxiety diagnoses similar to those found in our study. A U.S. study (Edelsohn et al., 2017) and a Finnish study (Varimo et al., 2020) found depression was the most frequent diagnosis associated with antipsychotics in 13‐17‐year‐olds, and a Norwegian study reported anxiety disorders and depression were the most common diagnoses in females 0‐18‐years (Nesvag et al., 2016). However, none of these studies used a hierarchical approach to associate diagnoses with prescriptions. It is possible that, for some patients, our diagnosis algorithm flagged comorbid depression/anxiety as the primary reason for antipsychotic prescribing when diagnoses higher in the hierarchy had not been captured in our dataset. However, our findings are consistent with international studies that used a range of techniques to link diagnoses with prescriptions, suggesting that the finding may be robust.

A Canadian study found GPs prescribed antipsychotics most often for depression and anxiety, whereas paediatricians prescribed them most often for ADHD, and child psychiatrists for psychotic disorders, in 2010/11 (Ronsley et al., 2013). Therefore, the high prescribing rates for depression/anxiety may indicate a prescribing pattern particular to general practice.

We also found increases in antipsychotic prescribing associated with bipolar disorder, DBD/CD, autism without behavioural problems, and eating disorders.

TGA approvals (Therapeutic Goods Administration, 2023) and guidelines for juvenile bipolar disorder (McClellan et al., 2007; Therapeutic Guidelines, 2021) recommend antipsychotics for treating manic symptoms. Therefore, the high percentage of patients (41.5% in 2017) with bipolar disorder prescribed antipsychotics is not surprising. However, bipolar disorder also had the greatest change (+19.9%) in prescribing from 2011 to 2017, which may indicate more liberal attitudes towards prescribing antipsychotics, or switching from epilepsy drugs such as valproate due to growing safety concerns (Medicines and Healthcare Products Regulatory Agency [MHRA], 2022).

More liberal prescribing practices may also apply to the other disorders showing an increase in prescribing. For example, the increase in prescribing for autism, without the behavioural problems required for on‐label prescribing, may indicate a growing belief that antipsychotics are suitable for a range of autistic symptoms. Alternatively, GPs may have omitted mention of behavioural issues in diagnostic fields, resulting in misclassification of some autism diagnoses.

The increased off‐label prescribing of antipsychotics for young patients with eating disorders is also concerning. Prescribing of medications to patients with eating disorders is problematic due to patients' poor nutritional status affecting drug metabolism and risk of adverse events (National Institute for Health and Care Excellence [NICE], 2017). The TGA has not approved antipsychotic use in eating disorders for any age‐group. Nonetheless, the advantage of weight gain as an effect of antipsychotic use in anorexia nervosa has led to the recommendation of antipsychotics, in particular olanzapine, as a treatment with some supportive evidence (Aigner et al., 2011), and the Royal Australian and New Zealand College of Psychiatrists clinical practice guidelines for eating disorders (Hay et al., 2014) document level‐II evidence supporting use of olanzapine and amisulpride.

### Sociodemographic characteristics of children/adolescents prescribed antipsychotics

Antipsychotic prescribing was more prominent in older age‐groups and increased monotonically with age, consistent with findings from 2007 to 2012 in the United States, United Kingdom, Denmark and Germany (Kalverdijk et al., 2017) and in Norway in 2010 (Nesvag et al., 2016).

The prescribing gap between the two genders decreased from 2011 to 2017 (male/female ratio declined from 1.8 to 1.5), and off‐label prescribing was higher in females than males in 2017. Supporting our findings, a recent Norwegian study found incident antipsychotic prescribing for females exceeded that in males for the first time in 2011, with the trend continuing through to 2017 (Varimo et al., 2020). The researchers speculated this might be due to increased antipsychotic prescribing for anxiety, mood disorders and insomnia, which are usually more prevalent in adolescent females. In line with this, in our study, the proportion of patients with depression/anxiety diagnoses prescribed antipsychotics was higher in 2017 than 2011.

With respect to socioeconomic factors, those in the most advantaged areas (highest quintile), were least likely to have antipsychotics associated with mental disorders. Although the proportion of patients with diagnoses prescribed antipsychotics generally increased as disadvantage increased, only those in the middle quintile were statistically significantly more likely to be prescribed antipsychotics for their diagnoses. Off‐label prescribing frequencies were similar across all quintiles in each year, except that they were markedly higher in the lowest quintile in 2017 compared with 2011. These results are suggestive of increased frequencies of prescribing to patients with increasing disadvantage, similar to that observed in U.K. (Nunn et al., 2020) and Swedish (Crump et al., 2011) studies. This may be due to poorer access to non‐pharmacological treatment options in socially disadvantaged areas (Nunn et al., 2020). However, the low numbers in our study did not permit analyses stratified by diagnosis and sociodemographics, limiting our understanding of the factors contributing to the observed pattern in antipsychotic prescribing. Future studies examining prescribing practices by age, rurality and socioeconomic status in more detail are needed.

### Choice of antipsychotic

The three most frequently prescribed antipsychotic agents were risperidone, quetiapine and olanzapine, consistent with findings in 11 of 14 countries (Hálfdánarson et al., 2017).

In the United States, several European countries, and Australia from 2005/6 to 2014 (Hálfdánarson et al., 2017), risperidone predominated. As in our study, international studies found risperidone prescribed mostly for hyperkinetic disorders (ADHD/CD) and autism (Bachmann et al., 2014; Murphy et al., 2013). However, in our study, risperidone was replaced by quetiapine in 2017 as the most frequently prescribed antipsychotic. This shift to quetiapine prescribing may be related to high prescribing rates for depression/anxiety, as observed in Finland from 2008 to 2017 (Varimo et al., 2020).

Notably, olanzapine, which was prescribed mainly for eating disorders, is not approved in Australia for any child or adolescent disorders. It is the antipsychotic agent most strongly associated with weight gain, and consequent cardiometabolic problems in young patients, putting paediatric patients at high risk of adverse events and long‐term harm (Maayan & Correll, 2011).

### Psychotropic co‐prescribing

Levels of concurrent psychotropic prescribing were high. In adjusted analyses, around 69% of patients were prescribed other psychotropics the same day as antipsychotics, in both 2011 and 2017. Corroborating our findings, a Finnish population‐wide prescription registry reported 44.9% of antipsychotic users from 2008 to 2016 had at least one concomitant psychotropic, although in their study polypharmacy rates were higher in 2016 (51.0%) than in 2008 (41.0%) (Varimo et al., 2023).

Consistent with Varimo et al. (2023), and a systematic review of antipsychotic polypharmacy in children/adolescents (primarily in the United States) (Toteja et al., 2014), we found antidepressants were most frequently co‐prescribed with antipsychotics. This was also congruent with findings from an earlier (2013) Pharmaceutical Benefits Scheme (PBS) report (Morrison et al., 2014), and a Canadian study characterising antipsychotic prescribing for young people up to 25 years from 2000 to 2007 (Murphy et al., 2013).

### Drivers of off‐label antipsychotic prescribing

In Australia, there are few reliable up‐to‐date resources to guide treatment of children/adolescents with psychotropic medications (Hazell, 2018), so clinicians must rely on alternative resources. These resources include consensus guidelines (Brett, 2015), adult guidelines (Doey et al., 2007), and international guidelines and approvals (Smogur et al., 2022). Prescribing patterns in our study may reflect advice provided in these resources.

For example, olanzapine is approved for adolescents with schizophrenia and bipolar disorder in the United States (Schulz & Haight, 2013), and for adults with these conditions in Australia (Eli Lilly & Co, 2019). Therefore, the relatively high prescribing in our study may be due to clinicians extrapolating medication effectiveness from these approvals. Similarly, quetiapine is TGA‐approved for depression and anxiety disorders in Australian adults (Brett, 2015), and appears to be increasingly prescribed for anxiety in children/adolescents (Monasterio & McKean, 2013). Dharni and Coates (2018) found quetiapine prescribed for anxiety (23.5%) and insomnia (76.5%) in 12‐17‐year‐olds—two conditions for which quetiapine is commonly prescribed for adults (Brett, 2015). Consistent with this, we found quetiapine was most frequently prescribed for depression/anxiety in both 2011 and 2017. Also, in the early 2000s, pharmaceutical companies in the United States actively promoted quetiapine for off‐label conditions in children/adolescents, such as anxiety, depression, PTSD and insomnia (U.S. Department of Justice, 2010). Such marketing campaigns can impact perceptions of safety and efficacy of medications, resulting in more liberal attitudes to prescribing, and increased off‐label prescribing (Fickweiler et al., 2017).

Increased off‐label prescribing of antipsychotics to children/adolescents in the United States has prompted health authorities to implement programs aiming to improve antipsychotic prescribing to children/adolescents in primary care (Penfold et al., 2022). One such programme resulted in a 51% relative decrease in antipsychotic prescribing from 2006 to 2013 (Barclay et al., 2017). Concerns about antipsychotic use in Australia prompted the Drug Utilisation Subcommittee (DUSC) of the PBS to prepare reports on antipsychotic prescribing in adults (NPS Medicine Wise Professional, 2020), and children/adolescents (Morrison et al., 2014). The report on adult prescribing resulted in the initiation of a programme providing GPs support and advice on managing patients with and without antipsychotics (NPS Medicine Wise Professional, 2020), resulting in a 7.3% relative decrease in antipsychotic prescribing. Results from our study suggest the need for similar strategies to improve antipsychotic prescribing in children/adolescents.

### Strengths and limitations

MedicineInsight is a large primary care database, covering 7% of GPs and 13% of patients who attended GPs in Australia in 2018/19. Although some states, regions, and higher IRSAD quintiles were over‐represented compared with census data, the patients in the database are considered representative of those attending Australian general practice (NPS MedicineWise, 2020a). MedicineInsight provides comprehensive data on medications prescribed, including both private and PBS‐subsidised prescriptions, and reasons for encounters. However, numbers were low in some analyses, limiting the reliability of some statistical findings.

Some patients had multiple diagnoses within a year, so it was not always possible to determine the exact diagnosis for which antipsychotics were prescribed. In some cases, terms used in the diagnosis algorithm represent both symptoms and diagnoses, which could result in overestimated prevalences of some common diagnoses, such as anxiety or behavioural disorders. Medications are often initiated by psychiatrists or paediatricians (Kjosavik et al., 2017), and some specialists' diagnoses might not be captured in our dataset. Similarly, diagnostic information in GP progress notes would not be captured, and historical information might not routinely be recorded at each clinical encounter. Also, anecdotal evidence suggests physicians may be reluctant to formally diagnose young patients, to reduce the risk of stigma (Olfson et al., 2015). Therefore, some diagnoses may be absent from MedicineInsight records, resulting in underestimates of the numbers of prescriptions associated with approved indications, and overestimates of off‐label prescribing. Furthermore, GP diagnoses may not match research criteria and may not be equivalent to diagnoses made by mental health specialists.

Medicine Insight records prescriptions written, not necessarily dispensed. Therefore, prescription frequencies may overestimate drug use. Finally, our findings only apply to general practice, and cannot be generalised to other settings.

## CONCLUSION

This study fills a gap in current understandings of antipsychotic prescribing to children/adolescents in Australia. Overall, the findings suggest an increase in GP off‐label prescribing from 2011 to 2017, especially for depression/anxiety.

There also appears to be an increasing tendency for patients in more disadvantaged areas to be prescribed antipsychotics for mental disorders, and a growing tendency to prescribe more to females. These changes in prescribing may be partly due to GPs having inadequate training and/or mentoring, and a lack of appropriate referral pathways. Future studies are needed to monitor antipsychotic prescribing trends, to assess the impact of COVID and other factors affecting mental health, and further investigate prescribing practices to paediatric patients in Australian primary care.

## AUTHOR CONTRIBUTIONS

**Julie Klau**: Conceptualization; formal analysis; funding acquisition; methodology; project administration; writing—original draft. **David Gonzalez**: Data curation; formal analysis; funding acquisition; methodology; supervision; writing—review and editing. **Melissa Raven**: Funding acquisition; methodology; supervision; writing—review and editing. **Jon Jureidini**: Conceptualization; funding acquisition; methodology; project administration; supervision; writing—review and editing.

## CONFLICT OF INTEREST STATEMENT

The authors have declared that they have no competing or potential conflicts of interest.

## ETHICAL CONSIDERATIONS

The independent MedicineInsight Data Governance Committee approved this study (protocol 2019‐029), and the Human Research Ethics Committee of the University of Adelaide exempted it from full ethical review due to the use of non‐identifiable data.

## Supporting information

Supporting Information S1

## Data Availability

The dataset used in this research is not publicly available due to privacy and ethical restrictions.

## APPENDIX A

Australia's health system

✓ Medicare—a universal publicly‐funded health insurance scheme available to all Australian and New Zealand citizens, permanent residents, and some overseas visitors
✓ Medicare Benefits Scheme (MBS) provides:
  ○ Low or no‐cost medical services, including general practitioner (GP) consultations, tests, scans
  ○ Free public hospital care
  ○ Subsidies for some services in private hospitals
✓ Pharmaceutical Benefits Scheme (PBS) (and the Repatriation PBS [RPBS] for eligible war veterans and their dependents) provides:
  ○ Low or no‐cost medicines for a large range of medicinal products that have been approved by Australia's regulatory authority ‐ the Therapeutic Goods Administration (TGA)
✓ MBS and PBS ‘safety net’ that annually caps out‐of‐pocket expenses for patients
✓ Private health insurance is incentivised by the Australian Government by providing a means‐tested rebate to assist with the cost of insurance.
✓ Private health insurance provides cover for private hospital patients and other health services not subsidised or partially subsidised by Medicare (e.g., dental, physiotherapy, non‐PBS‐subsidised medicines)

Mental health in primary care

✓ Mental health related issues are the most common reason for presentation to GPs
✓ GPs prescribe approximately 78% of all PBS‐subsidised antipsychotics
✓ GPs prescribe approximately 35% of PBS‐subsidised antipsychotics to children under 15 years
✓ GPs are the major providers of MBS‐subsidised mental health services
✓ Since 2006, Medicare has subsidised psychological therapies via the Better Access initiative (capped at 10 sessions annually)

For more information on Medicare: https://www.health.gov.au/topics/medicare/about.

For more information on the PBS: https://www.pbs.gov.au/info/about‐the‐pbs.
