# Supplementary material for: Antipsychotic prescribing patterns in children and adolescents attending Australian general practice in 2011 and 2017
Source: JCPP Adv. 2023 Nov 10;4(1):e12208. doi: 10.1002/jcv2.12208 (PMC10933664; doi:10.1002/jcv2.12208)
Supplement: Supplementary file 1 — Supporting Information S1 [file JCV2-4-e12208-s001.pdf]

Supporting Information

Article

Antipsychotic prescribing patterns in children and adolescents attending Australian general practice in 2011 and 2017.

Authors

Klau, J. Critical and Ethical Mental Health research group, Robinson Research Institute, Faculty of Health and Medical Sciences, The University of Adelaide, SA, Australia

Gonzalez-Chica, D. Discipline of General Practice, Faculty of Health and Medical Sciences, The University of Adelaide, SA, Australia

Raven, M. Critical and Ethical Mental Health research group, Robinson Research Institute, Faculty of Health and Medical Sciences, The University of Adelaide, SA, Australia

Jureidini, J. Critical and Ethical Mental Health research group, Robinson Research Institute, Faculty of Health and Medical Sciences, The University of Adelaide, SA, Australia

Contents

Table S1. Indications for use of antipsychotics in children and adolescents in Australia, as listed in Product Information documents approved by Australian Therapeutic Goods Administration 2

Table S2. Terms used to extract diagnostic information from MedicineInsight (diagnosis, reason for visit, reason for prescription) 3

Table S3. Reasons associated with antipsychotic prescriptions where there was no record of any psychiatric diagnosis 4

Table S4. Algorithm for determining off-label prescribing 5

Table S5. Changes in proportions of patients with diagnoses from 2011 to 2017, without hierarchy 7

Table S6. Number of patients co-prescribed other classes of psychotropics with an antipsychotic, by diagnosis with hierarchy 8

Table S7. Sensitivity analysis – Proportions of all children and adolescents with mental health diagnoses prescribed antipsychotics, 2011 and 2017 10

Table S1. Indications for use of antipsychotics in children and adolescents in Australia, as listed in Product Information documents approved by Australian Therapeutic Goods Administration

Medication	Medicine Name	Age range	Indicated use
**Antipsychotics**			
Chlorpromazine	Largactil	No age limits specified	Schizophrenia, mania, psychotic depression<br>Severe behavioural disturbances in children with mental retardation or autism: treatment of self-injurious, aggressive behaviour or overactivity<br>Nausea, vomiting
Clozapine	Clopine	> 16 years	Treatment resistant schizophrenia
Haloperidol	Serenace	> 2 years	Severely aggressive or hostile children, Tourettes
Lurasidone[a]	Latuda/Lupin	> 12 years	Schizophrenia
Periciazine	Neulactil	1-3 years	Not recommended – contraindicated < 1 year
Periciazine	Neulactil	3-6 years	Use reserved for exceptional situations in specialist units
Periciazine	Neulactil	> 6 years	Severe anxiety, impulsiveness, aggression, schizophrenia maintenance
Quetiapine	Seroquel	10-17 years	Monotherapy for treatment of acute mania associated with bipolar 1 disorder
Quetiapine	Seroquel	13-17 years	Schizophrenia
Risperidone	Risperdal	> 14 years	Schizophrenia
Risperidone	Risperdal	> 5 years	Treatment of conduct disorders or other disruptive behaviour disorders
Risperidone	Risperdal	Adolescents	Adolescents and adults with sub-average intellectual function or mental retardation and prominent destructive behaviours (e.g., aggression, impulsivity, self-injurious behaviours)
Risperidone	Risperdal	Children and adolescents	Behavioural disorders associated with autism in children and adolescents
Trifluoperazine	Stelazine[b]	>2 years (with caution)	Schizophrenia, manic depressive psychosis<br>Relief of delusions, hallucinations and confusion<br>Control of tremulousness and aggressive behaviour in alcoholics<br>Nausea and vomiting<br>Take particular care in administering Stelazine to children with acute illnesses (e.g. chicken pox, CNS infections, measles, gastroenteritis) or dehydration
Amisulpride	Solian		None
Aripiprazole	Abilify		None
Asenapine	Saphris		None
Brexpiprazole	Rexulti		None
Droperidol	Droleptan		Used only as injectable for anaesthesia in children 2 to 12 years
Flupenthixol	Fluanxol		None
Fluphenazine	Modecate		None
Olanzapine	Zyprexa		None
Paliperidone	Invega		None
Ziprasidone	Zeldox		None
Zuclopenthixol	Clopixol		None

a. Lurasidone was approved for schizophrenia in adolescents 13 to 17 years in April 2018

b. Stelazine is no longer registered on the Therapeutic Goods Administration (TGA) website. Last product information document updated 31 August 2016.

Table S2. Terms used to extract diagnostic information from MedicineInsight (diagnosis, reason for visit, reason for prescription)

Code	Diagnostic category	Relevant terms
SCHIZ	Psychoses, including schizophrenia	schizophrenia, schizoaffective, psychosis, delusions, hallucinations, paranoid, thought disorder, catatonic
BIP	Bipolar disorder	bipolar, manic depressive disorder, manic, mania, BPAD, bipolar affective disorder, mood swing
DBD	Disruptive behaviour disorder/ conduct disorder	CD, ODD, DBD, DMDD, disruptive, aggression, behaviour disorder, disruptive mood dysregulation disorder, defiance
ASD	Autism spectrum disorder	ASD, PDD, autism, asperger's, RETT syndrome
ADHD	Attention deficit hyperactivity disorder	ADHD, ADD, AHD, attention, hyperactive, hyperkinetic
ANX	Anxiety disorder	anxiety, anxious, phobia, GAD, generalised anxiety disorder, panic disorder/ panic attack, agoraphobia, neurotic
DEP	Depressive disorder	depression, dysthymia, melancholia, anhedonia, low moods, sad mood
ED	Eating disorder	anorexia, bulimia, binge eating disorder, body dysmorphia, orthorexia, PICA
PTSD	Post-traumatic stress disorder	PTSD, CPTSD, post-traumatic stress, complex trauma
OCD	Obsessive-compulsive disorder	OCD, obsessions, compulsions, trichotillomania,
PD	Personality disorder	PD, personality disorder, (antisocial, schizoid, schizoid, narcissistic, avoidant)
SUD	Substance use disorder	SUD, addiction, marijuana, cannabis, LSD, ecstasy, heroin, cocaine, MDMA
SLEEP	Sleep disorders, including insomnia	sleep, insomnia, narcolepsy, Willis-Ekbom, RLS, restless leg syndrome, hypersomnia
INCONT	Incontinence disorders, including enuresis	bed wetting, enuresis, incontinence
LD	Learning disorder	LD, learning disorder, dyslexia, dyscalculia, dysgraphia, reading problem,
LANG	Language disorder	language, apraxia, speech disorder

Note. After extracting diagnoses with key terms, all reasons from free text fields were manually checked to exclude non-diagnostic entries (e.g., “mother thinks child is….”; “without psychotic”).

Misspellings were also examined and included where appropriate (e.g., “austism”, “asbergers”, “dislexia”, “biopolar”, etc.).

Table S3. Reasons associated with antipsychotic prescriptions where there was no record of any psychiatric diagnosis

	2011		2017	
Reason associated with diagnosis	Number of patients	%	Number of patients	%
Tourettes	3	1.9	4	1.0
Epilepsy	6	3.9	7	1.8
Intellectual disability	2	1.3	3	0.8
Suicide/self-harm	1	0.6	4	1.0
Mental health care plans/ referrals	20	13.0	106	27.0
Vomiting/nausea	2	1.3	4	1.0
Repeat scripts	18	11.7	57	14.5
Infections	27	17.5	38	9.7
Skin conditions	3	1.9	6	1.5
Pain/migraine	3	1.9	6	1.5
Asthma	1	0.6	1	0.3
Allergy	1	0.6	2	0.5
Checks up/follow-up	2	1.3	13	3.3
Vaccination	0	0.0	6	1.5
Contraception/reproductive	1	0.6	4	1.0
Miscellaneous	64	41.6	131	33.4
**Total patients**	154	100.0	392	100.0

Note: Analysis of diagnoses using hierarchy. Diagnoses with TGA approval near top of hierarchy (Tourettes syndrome, epilepsy, nausea).

For all categories, the antipsychotic may have been given for a psychiatric reason that had not been recorded in our dataset. For patients with mental health plans, the diagnosis may be recorded in progress notes or specialist records. For other prescriptions, where there was no neurological or psychiatric reason on record, the reason for visit to the GP was recorded (e.g., infection).

Table S4. Algorithm for determining off-label prescribing

Diagnosis	On-label	Off-label
**Psychosis, including schizophrenia**	QUE + age >12 years	QUE + age<13 years
	RIS + age>14 years	RIS + age<15 years
	LUR + age>12 years	LUR + age<13 years
	PER + age>6 years	PER + age<7 years
	CLOZ + age>17 years	CLOZ + age<17 years
	TRIFLU + age>2 years	TRIFLU + age<3 years
	CHLOR	AMIS, ARIP, ASEN, BREX, DROP, FLUX, FLUZ, HAL, OLAN, PAL, ZIP, ZUC
**Autism with behaviour problems**	RIS, CHLOR	HAL + age<3 years
	HAL + age>2 years	PER + age<7 years
	PER + age>6 years	AMIS, ARIP, ASEN, BREX, CLOZ, DROP, FLUX, FLUZ, LUR, OLAN, PAL, QUE, TRIFLU, ZIP, ZUC
**Bipolar disorder[a]**	QUE + age>9 years	QUE + age<10 years
	CHLOR	AMIS, ARIP, ASEN, BREX, CLOZ, DROP, FLUX, FLUZ, HAL, LUR, OLAN, PAL, PER, RIS, TRIFLU, ZIP, ZUC
**Disruptive behaviour disorder/ conduct disorder**	RIS + age>5 years	RIS + age<6 years
	PER + age>6 years	PER + age<7 years
	HAL + age>2 years	HAL + age<3 years
	CHLOR	AMIS, ARIP, ASEN, BREX, CLOZ, DROP, FLUX, FLUZ, LUR, OLAN, PAL, QUE, TRIFLU, ZIP, ZUC
**Autism spectrum disorder**		AMIS, ARIP, ASEN, BREX, CHLOR, CLOZ, DROP, FLUX, FLUZ, HAL, LUR, OLAN, PAL, PER, QUE, RIS, TRIFLU, ZIP, ZUC
**Attention deficit hyperactivity disorder**		AMIS, ARIP, ASEN, BREX, CHLOR, CLOZ, DROP, FLUX, FLUZ, HAL, LUR, OLAN, PAL, PER, QUE, RIS, TRIFLU, ZIP, ZUC
**Eating disorders**		AMIS, ARIP, ASEN, BREX, CHLOR, CLOZ, DROP, FLUX, FLUZ, HAL, LUR, OLAN, PAL, PER, QUE, RIS, TRIFLU, ZIP, ZUC
**Depression or anxiety**	PER + age>6 years + diagnosis of anxiety	PER + age<7 years
		AMIS, ARIP, ASEN, BREX, CHLOR, CLOZ, DROP, FLUX, FLUZ, HAL, LUR, OLAN, PAL, QUE, RIS, TRIFLU, ZIP, ZUC
**Sleep problems**		AMIS, ARIP, ASEN, BREX, CHLOR, CLOZ, DROP, FLUX, FLUZ, HAL, LUR, OLAN, PAL, PER, QUE, RIS, TRIFLU, ZIP, ZUC
**Other[b]**		AMIS, ARIP, ASEN, BREX, CHLOR, CLOZ, DROP, FLUX, FLUZ, HAL, LUR, OLAN, PAL, PER, QUE, RIS, TRIFLU, ZIP, ZUC

Abbreviations for medications: AMIS, amisulpride; ARIP, aripiprazole; ASEN, asenapine; BREX, brexpiprazole; CHLOR, chlorpromazine; CLOZ, clozapine; DROP, droperidol; FLUX, flupenthixol; FLUZ, fluphenazine; HAL, haloperidol; LUR, lurasidone; OLAN, olanzapine; PAL, paliperidone; PER, periciazine; QUE, quetiapine; RIS, risperidone; TRIFLU, trifluoperazine; ZIP, ziprasidone; ZUC, zuclopenthixol

a. Antipsychotics are approved for children/adolescents only for manic episodes within bipolar 1 disorder. For the purposes of this algorithm, any prescription of an antipsychotic with bipolar disorder was assumed to be for mania, and therefore, on-label.

b. Other diagnoses include obsessive-compulsive disorder, post-traumatic stress disorder, personality disorder, substance use disorder, incontinence disorders (including enuresis/bed wetting), learning disorder, language disorder.

Table S5. Changes in proportions of patients with diagnoses from 2011 to 2017, without hierarchy

	Predicted % 2011	Predicted % 2017	Difference 2011 to 2017 (95% CI)[a]	p value
Any mental health diagnosis	4.22	6.74	2.52 (2.21, 2.83)	<0.001
Psychosis	0.03	0.05	0.02 (0.01, 0.03)	0.001
Bipolar disorder	0.04	0.04	-0.001 (-0.02, 0.01)	0.550
ASD	0.63	0.82	0.20 (0.12, 0.27)	<0.001
DBD/CD	0.47	0.72	0.25 (0.18, 0.32)	<0.001
ADHD	0.55	1.12	0.57 (0.44, 0.70)	<0.001
ED	0.09	0.13	0.04 (0.02, 0.06)	<0.001
Depression	0.83	1.29	0.45 (0.38, 0.53)	<0.001
Anxiety	0.92	2.17	1.25 (1.14, 1.37)	<0.001
Sleep	0.44	0.95	0.51 (0.42, 0.60)	<0.001
OCD	0.08	0.09	0.01 (-0.01, 0.03)	0.186
PTSD	0.03	0.05	0.02 (0.01, 0.04)	<0.001
PD	0.02	0.02	0.004 (-0.005, 0.01)	0.375
SUD	0.06	0.06	-0.00 (-0.02, 0.02)	0.990
Incontinence/enuresis	0.39	0.40	0.01 (-0.03, 0.05)	0.726
Learning disorder	0.09	0.10	0.02 (-0.004, 0.04)	0.117
Language disorder	0.36	0.55	0.19 (0.13, 0.25)	<0.001

Abbreviations: ASD, autism spectrum disorder; DBD, disruptive behaviour disorder; CD, conduct disorder; ADHD, attention deficit hyperactivity disorder; ED, eating disorder; OCD, obsessive-compulsive disorder; PTSD, post-traumatic stress disorder; PD, personality disorder; SUD, substance use disorder

a. Analyses using logistic regression, adjusted for age, gender, patient IRSAD, and practice characteristics (state, rurality, practice IRSAD). IRSAD, Index of Relative Socio-economic Advantage and Disadvantage.

Table S6. Number of patients co-prescribed other classes of psychotropics with an antipsychotic, by diagnosis with hierarchy

	2011			2017		
Diagnoses	AP (N)	Any concurrent n (%)[a]	Most frequent concurrent (n[%])[a]	AP (N)	Any concurrent n (%)[a]	Most frequent concurrent (n[%])[a]
All	191	132 (69.1)	ADEP(108 [56.54])	893	660 (73.9)	ADEP (511[57.22])
			ADHD (24[12.57])<br>STIM(17[8.90])<br>ADREN(11[5.76])			ADHD (186[20.83])<br>STIM(135[15.12])<br>ADREN(92[10.30])
			ANXIO (24[12.57])			MEL (139[15.57])
Psychoses, including schizophrenia	33	27 (81.82)	ADEP (25[75.76])	81	45 (55.56)	ADEP (39[48.15])
			ANXIO (8[24.24])			ANXIO (9[11.11])
			SED <5			MEL (8[9.88])
Autism with behavioural problems	7	2 (28.57)	ADHD <5	23	14 (60.87)	ADEP (10[43.48])
			ANXIO <5			ADHD (6[26.09])<br>STIM(6[26.09])<br>ADREN <5
						MEL (2[8.70])
Bipolar disorder	21	12 (57.14)	ADEP (11[52.38])	66	45 (68.18)	ADEP (39[59.1])
			ANXIO (94[19.05])			ADHD (7[10.61])<br>STIM(6[9.09])<br>ADREN <5
			SED <5			ANXIO&SED(6[9.09])
Disruptive behaviour/conduct disorders	13	8 (61.54)	ADEP (6[46.15])	76	51 (67.11)	ADHD (30[39.47])<br>STIM (19[25.0])<br>ADREN (21[27.63])
			ADHD (5[38.46])<br>STIM <5<br>ADREN <5			ADEP (24[31.58])
			ANXIO <5			MEL (13[17.11])
Autism spectrum disorder	44	24 (54.55)	ADEP (13[29.55])	206	134 (65.05)	ADEP (84[40.78])
			ADHD (12[27.27])<br>STIM(8[18.18])<br>ADREN(5[11.36])			ADHD (66[32.04])<br>STIM(51[24.76])<br>ADREN(27[13.11])
			MEL <5			MEL (25[12.14])
Attention deficit hyperactivity disorder	10	5 (50.0)	ADHD <5	78	60 (76.92)	ADHD (51[65.38])<br>STIM (45[57.7])<br>ADREN (20[25.6])
			ADEP <5			ADEP (29[37.18])
						MEL (5[6.41])
Eating disorders	<5	<5	ADEP <5	34	30 (88.24)	ADEP (28[82.35])
						MEL (4[11.76])
						ANXIO <5
Depression/anxiety	50	42 (84.0)	ADEP (41[82.0])	300	263 (87.67)	ADEP (249[83.0])
			ANXIO (7[14.0])			MEL (67[22.33])
			SED <5			ANXIO (36[12.0])
Sleep	7	7 (100.0)	ADEP (5[71.4])	14	11 (78.57)	MEL (7[50.0])
			MEL <5			ADEP <5
			ANXIO&SED <5			ADHD <5
Other[b]	<5	<5	ADEP <5	15	7 (46.67)	ADEP (5[33.33])
						SED&MEL <5
						ADHD <5

Note: Numbers and percentages were omitted where cell size <5 patients

Abbreviations: AP, antipsychotic; ADEP, antidepressant; ANXIO, anxiolytic; ADHD, medication for attention deficit hyperactivity disorder, including stimulants and alpha2 agonists; STIM, stimulant; ADREN, alpha2 agonist; SED, hypnotic/sedative; RIS, risperidone; QUE, quetiapine; OLAN, olanzapine; ARIP, aripiprazole; HAL, haloperidol; PER, periciazine; MEL, melatonin.

a. Percentages reported as unadjusted proportion of all patients on antipsychotics for each diagnostic group. Patients may have had more than one co-prescribed medication class.
b. 'Other' includes obsessive-compulsive disorder, post-traumatic stress disorder, personality disorder, substance use disorder, incontinence disorder, including bed-wetting, learning and language disorders

Table S7. Sensitivity analysis – Proportions of all children and adolescents with mental health diagnoses prescribed antipsychotics, 2011 and 2017

	2011		2017		Absolute difference 2017 -2011	
	N with diagnosis	Adjusted[a] % of diagnostic group receiving antipsychotic prescriptions	N with diagnosis	Adjusted[a] % of diagnostic group receiving antipsychotic prescriptions	%(95% CI)	p value
**All diagnoses***	8702	1.8	28814	2.8	0.9 (0.5, 1.4)[a]	<0.001
**Psychoses, including schizophrenia**	70	40.3	218	31.2	-9.1 (-22.5, 4.3)	0.183
**Autism with behavioural problems**	66	10.3	250	8.4	-1.9 (-8.8, 5.0)	0.584
**Bipolar disorder**	85	19.6	161	40.1	20.5 (9.6, 31.4)	<0.001
**Disruptive behaviour/ conduct disorder**	874	1.2	2943	2.9	1.7 (0.5, 3.0)	0.008
**Autism without behavioural problems**	1158	3.5	3291	5.9	2.3 (0.2, 4.4)	0.029
**ADHD**	933	0.8	3759	1.7	0.9 (0.2, 1.6)	0.015
**Eating disorders**	187	1.0	543	6.5	5.5 (2.7, 8.3)	<0.001
**Depression or anxiety**	2956	1.4	10642	2.4	1.0 (0.5, 1.6)	<0.001
**Depression**	1680	1.7	4941	3.7	1.9 (1.2, 2.7)	<0.001
**Anxiety**	1805	1.2	8110	2.1	0.9 (0.3, 1.5)	0.005
**Depression and anxiety**	529	1.7	2409	4.1	2.4 (1.2, 3.6)	<0.001
**Sleep disorders**	741	0.8	3359	0.6	-0.2 (-0.9, 0.4)	0.467
**Other mental health[b]**	1632	0.2	4250	0.4	0.2 (-0.1, 0.5)	0.113

Note. Analysis including all children and adolescents attending general practices, not restricted to 'regular patients' (i.e., patients with visits 3 times within 2 years, and 1 visit in each year)
* Only one diagnosis is recorded per patient according to hierarchy. Highest level (1: psychosis) through to lowest level (10: other). Diagnoses higher in hierarchy have stronger clinical rationale for use of antipsychotics. Numbers adjusted for age, gender, patient IRSAD, and practice characteristics (state, rurality, practice IRSAD).
Abbreviations: ADHD, attention deficit hyperactivity disorder.
a. Analysis using logistic regression adjusted for age, gender, patient IRSAD, and practice characteristics (state, rurality, practice IRSAD). IRSAD, Index of Relative Socio-economic Advantage and Disadvantage.
b 'Other' includes obsessive-compulsive disorder, post-traumatic stress disorder, personality disorder, substance use disorder, incontinence disorder, including bedwetting, learning and language disorders.
